# The association between periconceptional folic acid supplementation and the risk of preterm birth: a population-based retrospective cohort study of 200,000 women in China

**DOI:** 10.1007/s00394-020-02409-8

**Published:** 2020-10-19

**Authors:** Yu Wu, Yanling Yuan, Cai Kong, Qiuyue Ma, Hanfeng Ye, Wenzhan Jing, Jue Liu, Min Liu

**Affiliations:** 1 Department of Epidemiology and Biostatics, School of Public Health, Peking University, No.38, Xueyuan Road, Haidian District, Beijing, 100191 China; 2 Yunnan Population and Family Planning Research Institute, No.146, Qingnian Road, Wuhua District, Kunming, 650021 Yunnan China

**Keywords:** Periconception, Folic acid supplementation, Preterm birth, China

## Abstract

**Purpose:**

The aim of this study was to examine the association between periconceptional folic acid (FA) supplementation and risk of preterm birth.

**Methods:**

We conducted a retrospective cohort study in women aged 18–49 who participated in the NFPHEP from 2010 to 2018, and had a singleton livebirth in 129 counties in southwest China. Participants were divided into four groups according to the time period starting FA use: no use, after the last menstrual period, at least 1–2 months before the last menstrual period, at least 3 months before the last menstrual period. The outcomes were preterm birth (gestation < 37 weeks) and early preterm birth (gestation < 34 weeks).

**Results:**

201,477 women were included and 191,809 (95.2%) had taken FA during periconception. Compared with women who did not take FA, women who started taking FA 1–2 months before their last menstrual period had a 15% lower risk of preterm birth (aOR = 0.85, 95% CI 0.79–0.92), and women who started taking FA at least 3 months before their last menstrual period had a 20% lower risk of preterm birth (aOR = 0.80, 95% CI 0.75–0.87), but women who started taking FA after their last menstrual period did not appear to reduce the risk of preterm birth.

**Conclusions:**

In this study of 200,000 Chinese women, periconceptional supplementation with FA was associated with a lower risk of preterm birth. Women who started taking FA at least 3 months before their last menstrual period were more likely to reduce the risk of preterm birth.

## Background

Folic acid (FA) is a water-soluble member of the vitamin B complex family, and it has an essential role in DNA methylation and synthesis [1, 2]. Previous studies have shown that fortification of nutrients with FA and FA supplementation is an important measure to improve the outcome of pregnancy [3]. FA has a preventive effect on the occurrence of neural tube defects (NTDs) which has been proved by large randomized trials [4–6]. FA supplementation during periconception may decrease the risk of pre-eclampsia, miscarriage, low birth weight, small for gestational age, stillbirth, neonatal death and autism in children [7]. However, the association between FA supplementation during periconception and other adverse pregnancy outcomes is still unclear.

Preterm birth complications are the leading cause of death among children under 5 years of age [8]. Approximately 1 million children under 5 years die each year due to complications of preterm birth, accounting for 17.8% of under-5 deaths [8, 9]. About 944,000 neonates died from preterm birth, accounting for 35.3% of all neonatal deaths [9]. In addition, preterm birth may increase risks of neonatal respiratory diseases (such as respiratory distress syndrome and bronchopulmonary dysplasia), necrotizing enterocolitis, sepsis, neurological conditions (such as periventricular leukomalacia, seizures, intraventricular hemorrhage, cerebral palsy, and hypoxic ischemic encephalopathy) [10], as well as feeding difficulties and visual and hearing disorders [11, 12]. In the long term, preterm birth has been linked to behavioral, social–emotional, and learning difficulties in childhood [13], which causes psychological and economic burdens for the families of premature neonates.

With a large population and a large number of births, China has the second greatest number of preterm births in the world [8], so addressing preterm birth is critical to addressing neonatal and child mortality and morbidity. To improve the health of women of childbearing age and reduce birth defects, the Chinese central government launched the Health System Reform Plan. Periconceptional FA supplementation in rural Chinese women was included in a package of major public health services [14]. The national program proposal for FA supplementation suggested that a daily dose of one 0.4 mg FA tablet would be used for 3 months before pregnancy to their first trimester of pregnancy for the pre-pregnancy women [15]. From 2009 to 2010, the Chinese central government invested 190 million renminbi (RMB) to provide FA supplementation for a total of 13.18 million women of childbearing age in rural areas of China to prevent birth defects [16]. According to the National Annals of Statistics of China from 2010 to 2018, about 148.81 million babies were born in China, which means about 140 million women receive free prenatal FA supplements from the government [17]. We did a large population-based retrospective cohort study in women of childbearing age in Southwest China to examine the association between periconceptional FA supplementation and risk of preterm birth systematically so as to evaluate the effect of periconceptional FA supplementation on reducing preterm birth.

## Methods

### Data sources

We did a population-based retrospective cohort study in women of childbearing age (18–49 years) who participated in the National Free Preconception Health Examination Project (NFPHEP) from Jan 1, 2010, to Dec 31, 2018, and had a singleton livebirth in 129 counties in southwest China. NFPHEP was launched by the Chinese National Health and Family Planning Commission in 2010. It aims to provide free health examinations and other services before conception for couples who planned to become pregnant in the next 6 months, as well as follow-up in their first trimester of pregnancy and after delivery. This study was approved by the Institutional Review Board of the Chinese Association of Maternal and Child Health Studies. All participants provided written informed consent before enrolment [18].

### Procedures

Trained qualified local health workers registered couples of childbearing age who were planning to become pregnant in the next 6 months and used the standardized questionnaire to collect baseline information from women of childbearing age, including sociodemographic characteristics (age, ethnicity, education level, occupation); history of pregnancy and adverse pregnancy outcomes (gravidity, parity, history of abortion, history of preterm birth, history of stillbirth); history of chronic disease (hypertension, heart disease, diabetes, chronic nephritis, thyroid disease, and cancer). Health workers measured height and weight of participants and calculated the body mass index (BMI) by dividing the weight in kg by the square of the height in m. Meanwhile, venous blood was extracted from the participants by qualified and trained professionals, and serum samples were separated and tested in the laboratories of accredited medical institutions. The concentration of hemoglobin was measured using the cyanide methemoglobin method. In this study, the diagnostic criteria for anemia referred to the diagnostic threshold recommended by WHO (anemia was defined as lower than 120 g/L for non-pregnant women) and adjusted according to altitude [19]. All samples were tested for the presence of hepatitis B surface antigen (HBsAg) using ELISA kits. HBsAg positivity indicated that a participant was infected with hepatitis B virus.

Maternal and child health workers interviewed participants face-to-face or by telephone in 3 months after conception, recording their last menstrual period, their supplemental use of FA, their living habits of eating meat and eggs, eating vegetables, smoking, and drinking alcohol during pregnancy. All participants were followed up by health workers for 1 month after delivery to collect information including pregnancy outcome (normal birth, preterm birth, abortion, or stillbirth), delivery date, gestational weeks, and newborn information (singleton or multiple births). The study was terminated when participants had preterm birth or other pregnancy outcomes, or when the study reached the end of the observation period (Dec 31, 2018).

### Exposure

In this study, we defined the use of FA supplement as taking 1 tablet (0.4 mg) of FA a day. Participants were divided into four groups according to the time starting FA use: no use (control group), after the last menstrual period (exposure group I), at least 1–2 months before the last menstrual period (exposure group II), at least three months before the last menstrual period (exposure group III).

### Outcomes

The primary outcome was preterm birth which was defined as a delivery from 28 weeks to less than 37 weeks of gestation. Early preterm birth was defined as a delivery from 28 weeks to less than 34 weeks of gestation. Preterm and early preterm birth rates were the proportion of premature and early premature births in the total number of all singleton livebirths, respectively.

### Statistical analysis

We included all women who fitted all inclusion criteria, and described the distributions of FA using time of the participants in different sociodemographic characteristics (age, ethnicity, education level, occupation), history of pregnancy and adverse pregnancy outcomes (gravidity, parity, history of preterm birth, history of abortion, history of stillbirth), physical condition (BMI, anemia, infection of hepatitis B), living habits during pregnancy (eating meat and eggs, eating vegetables, smoking, drinking alcohol). The χ^2^ test was used for inter-group comparison.

Our study used the univariate logistic model to obtain crude odds ratio (cOR), and used multivariate logistic model to adjust the potential risk factors for preterm birth and obtained adjusted odds ratio (aOR) and 95% confidence intervals (95% CI) for FA supplementation and preterm, early preterm birth rates. To test the robustness of the results, we adjusted different covariates in stages. In model A, we adjusted for sociodemographic characteristics of participants, including age (18–20 years, 21–25 years, 26–30 years, 31–35 years, or 36–49 years); ethnicity (Han, minorities including Yi, Dai, Miao, Hani and others); level of education (primary school or below, junior high school, senior high school, or college or higher); and occupation (farmers, workers, or others). In model B, in addition to those factors included in model A, the history of pregnancy and history of adverse pregnancy outcomes were also adjusted, including first gestation (yes or no), primipara (yes or no), history of preterm birth (yes or no), history of abortion (yes or no), and history of stillbirth (yes or no). In model C, in addition to those factors included in model B, we also adjusted for physical conditions of participants, including BMI (< 18.5 kg/m^2^, 18.5–23.9 kg/m^2^, 24.0–27.9 kg/m^2^, or ≥ 28.0 kg/m^2^), anemia (yes or no), and HBsAg (positive or negative). In model D, we additionally adjusted for living habits during pregnancy, including eating meat and eggs (yes or no), eating vegetables (yes or no), smoking (yes or no), and drinking alcohol (yes or no). Then the adjusted ORs and 95% CIs for supplementation with FA and preterm, early preterm birth were obtained.

In the subgroup analysis, we divided the participants into different subgroups according to the baseline characteristics. In different subgroups, we adjusted for other potential risk factors for preterm birth to find an association between preterm birth and FA supplementation in women. All of the analyses were done with SPSS 21 software. Two-sided *p* values of less than 0.05 were considered statistically significant.

## Results

**Fig. 1:**
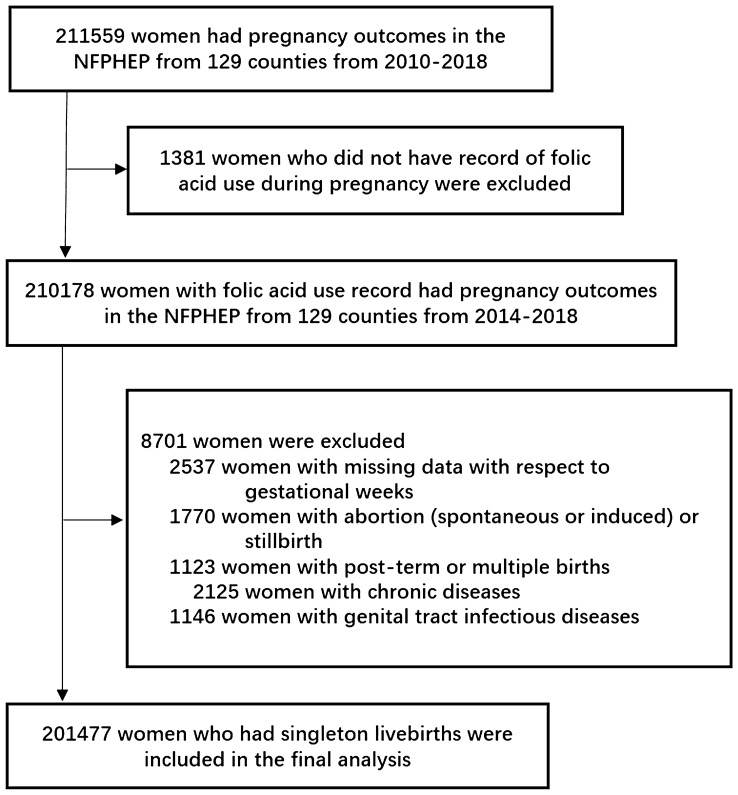
Inclusion and exclusion criteria of participants. NFPHEP National Free Preconception Health Examination Project

From January 1, 2010 to December 31, 2018, 211,559 women had delivery record. We excluded 1381 women who did not have record of FA use during pregnancy; 2537 women with missing data on gestational weeks; 1770 women with abortion or stillbirth; 1123 women with post-term or multiple births; 2125 women with chronic diseases (including hypertension, heart disease, diabetes, chronic nephritis, thyroid disease, and cancer); 1146 women with genital tract infectious disease. 201,477 women were included in the final study (Fig. 1).

Among the 201,477 women of childbearing age, 9668 women did not take FA during periconception and 191,809 (95.2%) had taken FA during periconception. Women aged 18–20 years, of Miao, Hani ethnicity and other ethnic minorities, with primary education and below, with BMI ≥ 28.0 kg/m^2^, as well as women who were anemic and did not eat vegetables were less likely to take FA (Table 1).

**Table 1:** Distribution of the starting time of FA supplementation in women with different characteristics

	Total (*n*)	The starting time of folic acid supplementation (%)				*χ*^2^	Preterm birth (%)
		No supplementation	After the last menstrual period	1–2 months before the last menstrual period	3 months before the last menstrual period		
Total	201,477	9668 (4.8)	35,025 (17.4)	52,408 (26.0)	104,376 (51.8)		15,850 (7.9)
**Age**						< 0.001	
18–20	18,028	7.1	21.1	27.2	44.6		9.7
21–25	80,431	4.7	18.2	26.3	50.8		7.9
26–30	69,717	4.2	16.3	25.5	54.0		7.3
31–35	24,176	5.0	15.8	25.8	53.4		7.7
36–49	9125	5.5	15.9	24.8	53.8		8.8
Ethnicity*						< 0.001	
Han	124,442	4.1	16.9	24.9	54.1		7.2
Minority	76,708	5.9	18.2	27.9	48.0		8.9
Yi	34,381	4.7	18.7	31.1	45.5		6.8
Dai	8647	4.9	16.3	23.4	55.4		10.8
Miao	5736	6.2	20.9	34.9	38.0		12.7
Hani	5462	8.7	19.7	24.4	47.2		8.0
Other	22,482	7.3	17.1	23.8	51.7		10.8
Education*						< 0.001	
Primary school or below	41,443	6.3	17.4	28.7	47.6		8.9
Junior high school	108,861	4.5	17.4	25.8	52.3		7.9
Senior high school	27,207	4.4	18.5	25.0	52.0		7.0
College or higher	21,380	4.2	15.8	23.6	56.4		6.5
**Occupation**						< 0.001	
Worker	9306	4.3	17.9	23.9	53.8		7.0
Farmer	184,592	4.9	17.2	26.2	51.7		7.9
Other	7579	3.6	20.0	23.7	52.8		7.6
**History of pregnancy and adverse pregnancy outcomes**							
First gestation*	79,158	4.1	17.8	25.4	52.6	< 0.001	8.0
Primipara	97,925	4.4	18.6	25.9	51.2	< 0.001	7.9
History of preterm birth	751	5.6	17.7	28.9	47.8	0.123	15.4
History of abortion	52,446	5.6	19.4	28.4	46.7	< 0.001	7.6
History of stillbirth	1730	5.7	17.5	26.4	50.4	0.275	9.1
** Physical condition**							
Body mass index (kg/m^2^)*						< 0.001	
< 18.5	26,533	4.4	18.2	26.1	51.3		8.2
18.5–23.9	143,132	4.7	17.1	26.1	52.1		7.7
24.0–27.9	25,763	5.6	17.7	25.7	51.1		8.1
≥ 28.0	5712	6.2	18.4	24.8	50.6		8.8
Anemia*	36,147	6.2	19.3	26.5	48.1	< 0.001	8.6
HBsAg positive	4904	5.6	18.7	26.5	49.3	< 0.001	8.3
**Living habits during pregnancy**							
Eating meat and eggs*	195,671	4.7	17.3	26.1	51.8	< 0.001	7.9
No eating vegetables*	5522	6.5	17.7	24.2	51.6	< 0.001	9.7
Smoking*	3589	3.2	11.8	27.8	57.2	< 0.001	10.1
Drinking alcohol*	4108	3.1	13.7	27.4	55.9	< 0.001	10.0

*Denominators provided were some data were missing

Of the 201,477 women, 15,850 had preterm birth, with the preterm birth rate of 7.9% (95% CI 7.8–8.0%). Univariate regression showed that the starting time of FA supplementation in women was associated with preterm birth (*P* < 0.05). Multivariate logistic models showed that after adjusting for covariates, compared with women who did not take FA, women who started taking FA 1–2 months before their last menstrual period had a 15% lower risk of preterm birth, and women who started taking FA at least 3 months before their last menstrual period had a 20% lower risk of preterm birth. After adjusting for different variables, the aORs obtained by models A, B, C and D were relatively robust (Table 2).

**Table 2:** Association between the starting time of FA supplementation and preterm birth after adjusting for different covariates

	The time of folic acid supplementation			
	No supplementation (*n* = 9668)	After the last menstrual period to the end of the first trimester (*n* = 35,025)	1–2 months before the last menstrual period to the end of the first trimester (*n* = 52,408)	3 months before the last menstrual period to the end of the first trimester (*n* = 104,376)
**Preterm birth% (95% CI)**	9.4 (8.8, 10.0)	8.7 (8.5, 9.0)	7.9 (7.7, 8.2)	7.4 (7.2, 7.6)
Crude OR (95% CI)	1.00	0.92 (0.85, 1.00)	0.83 (0.77, 0.89)	0.77 (0.71, 0.82)
Adjusted OR (95% CI)*				
Model A	1.00	0.95 (0.87, 1.02)	0.85 (0.79, 0.92)	0.80 (0.75, 0.87)
Model B	1.00	0.94 (0.87, 1.02)	0.85 (0.78, 0.91)	0.80 (0.74, 0.86)
Model C	1.00	0.95 (0.88, 1.03)	0.85 (0.79, 0.92)	0.81 (0.75, 0.87)
Model D	1.00	0.95 (0.88, 1.03)	0.85 (0.79, 0.92)	0.80 (0.75, 0.87)
**Early preterm birth% (95% CI)**	3.6 (3.3, 4.0)	3.1 (2.9, 3.3)	2.9 (2.7, 3.0)	2.7 (2.6, 2.8)
Crude OR (95% CI)	1.00	0.83 (0.73, 0.94)	0.77 (0.68, 0.87)	0.72 (0.64, 0.80)
Adjusted OR (95% CI)*				
Model A	1.00	0.86 (0.76, 0.97)	0.80 (0.71, 0.90)	0.76 (0.68, 0.85)
Model B	1.00	0.87 (0.77, 0.98)	0.81 (0.72, 0.91)	0.77 (0.69, 0.86)
Model C	1.00	0.86 (0.76, 0.98)	0.81 (0.71, 0.91)	0.77 (0.68, 0.86)
Model D	1.00	0.86 (0.76, 0.98)	0.81 (0.72, 0.91)	0.77 (0.69, 0.86)

*Model A adjusted for sociodemographic characteristics (age, ethnicity, education level, occupation)

Model B adjusted for those factors included in model A and history of pregnancy and adverse pregnancy outcomes (first gestation, primipara, history of preterm birth, history of abortion, history of stillbirth)

Model C adjusted for those factors included in model B and physical conditions (BMI, anemia, HBsAg)

Model D adjusted for those factors included in model C and living habits during pregnancy (eating meat and eggs, eating vegetables, smoking, drinking alcohol)

Of the 201,477 women, 5,706 showed early preterm birth (gestation at less than 34 weeks). Women who did not take FA, who started taking FA after their last menstrual period, who started taking FA 1–2 months before their last menstrual period, and who started taking FA at least 3 months before their last menstrual period had an early preterm birth rate of 3.6%, 3.1%, 2.9% and 2.7%, respectively, with a significant decrease (*χ*_trend_^2^ = 39.34, P < 0.001). Multivariate logistic models showed that after adjusting for covariates, women who had taken FA during periconception had a lower risk of early preterm birth than women who did not take FA, with significant results (*P* < 0.05). The adjustment of different covariables did not affect the results, and the results of the four models were robust (Table 2).

**Fig. 2:**
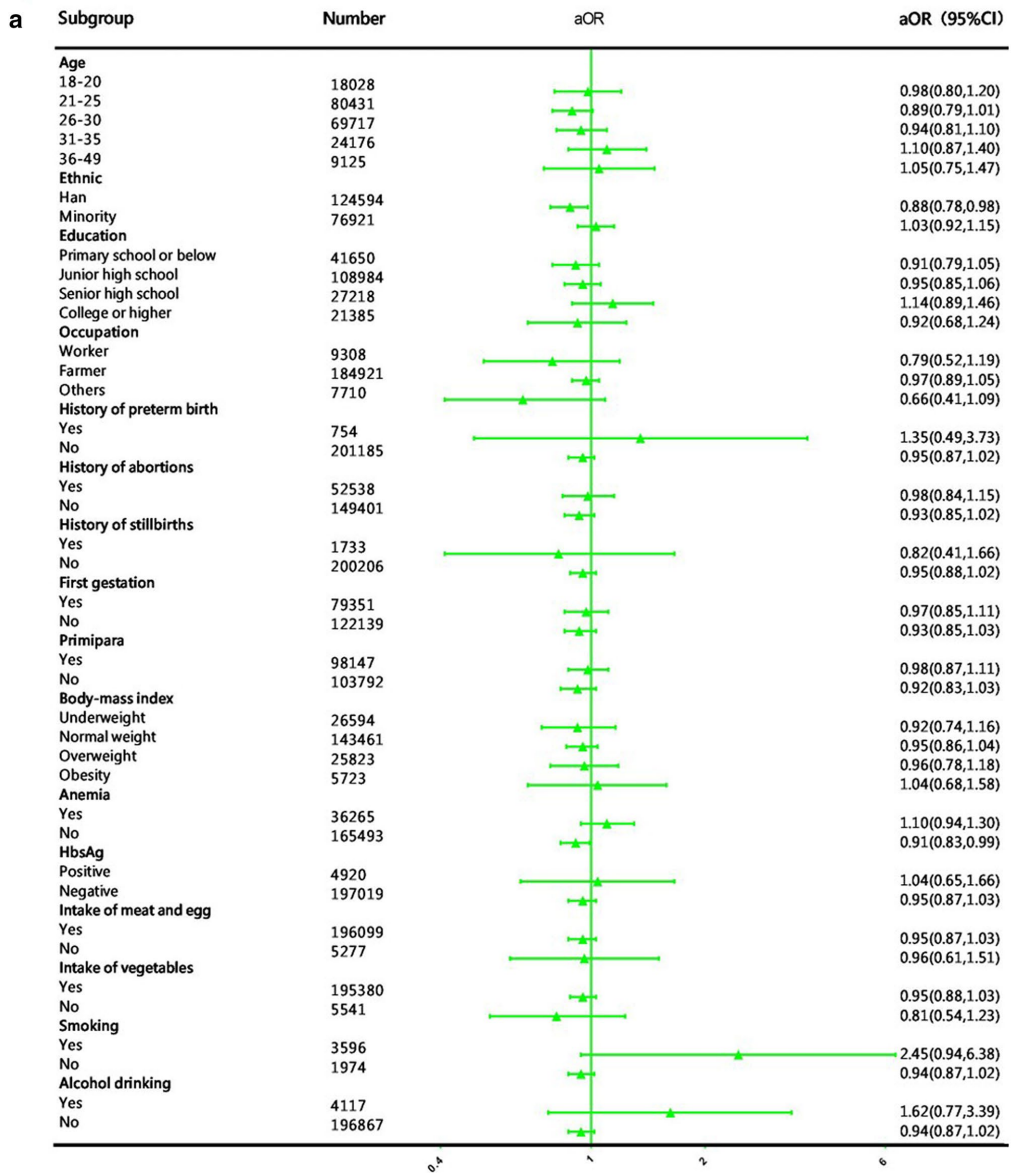

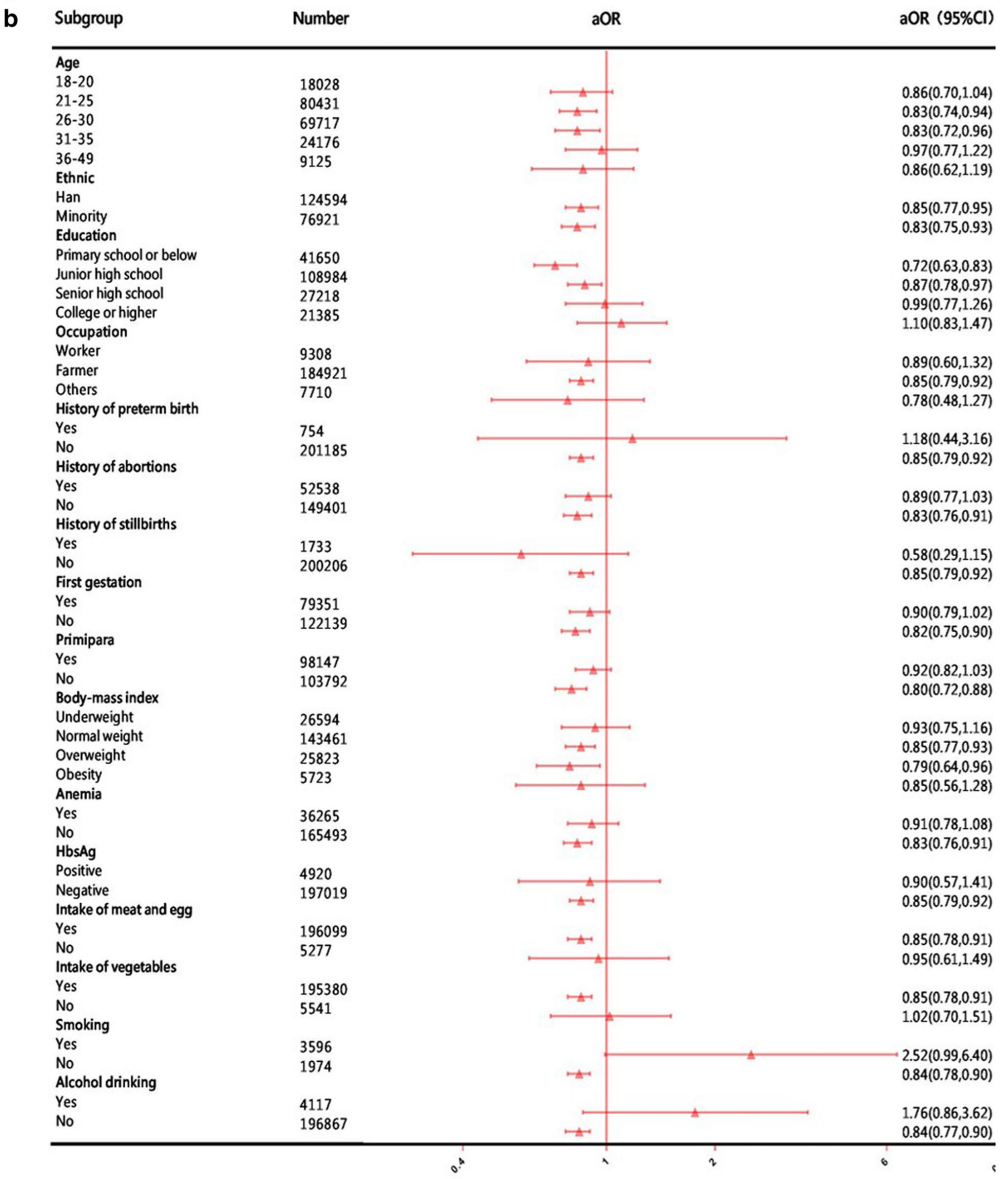

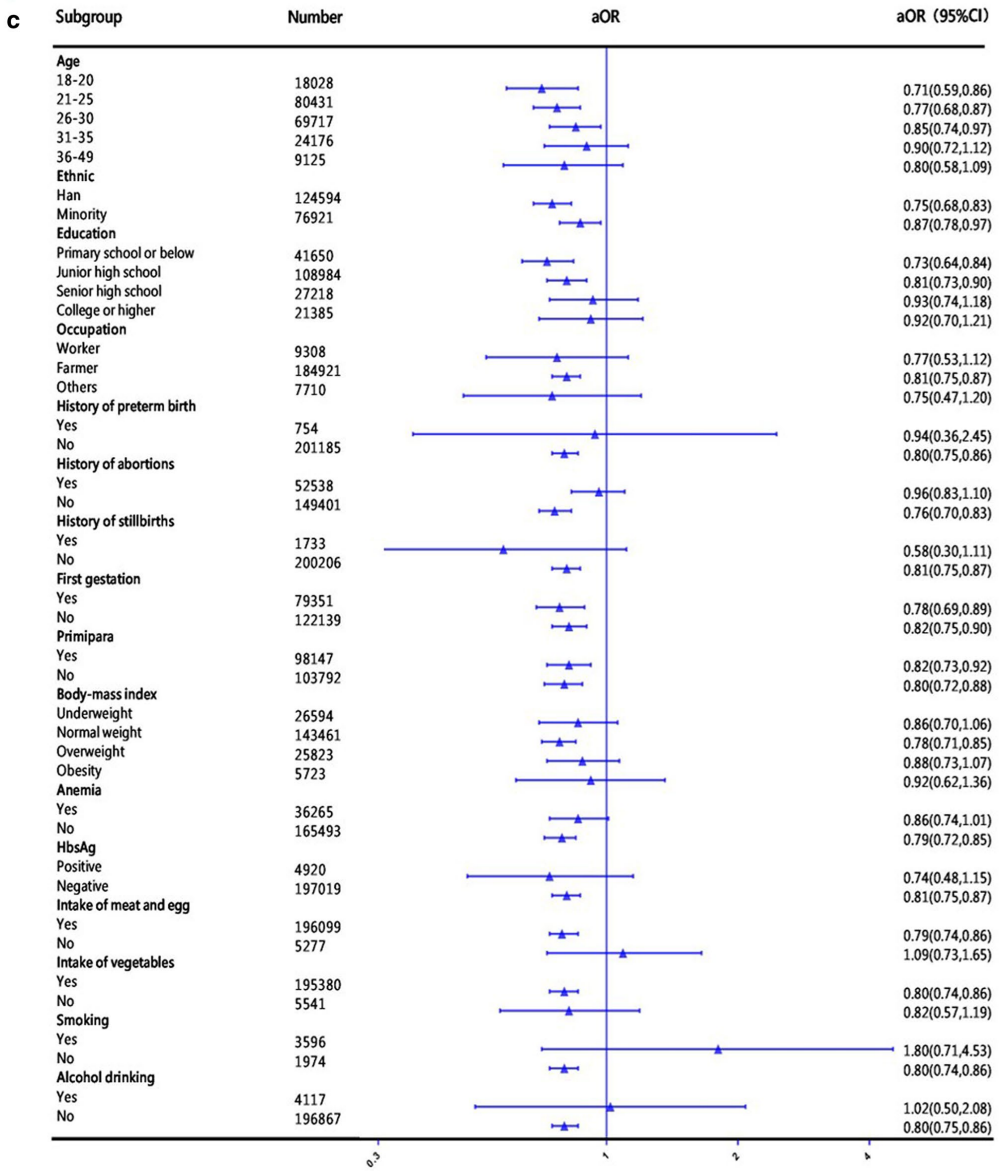
Subgroup analysis of women's FA supplementation associated with preterm birth. **a** Subgroup analysis of women's FA supplementation after the last menstrual period associated with preterm birth compared with women who did not take FA. **b** Subgroup analysis of women's FA supplementation 1–2 months before their last menstrual period associated with preterm birth compared with women who did not take FA. **c** Subgroup analysis of women's FA supplementation at least 3 months before their last menstrual period associated with preterm birth compared with women who did not take FA

In the sensitivity analyses, the associations between the time of FA supplementation in periconceptional women and preterm birth did not change significantly in different baseline characteristics (sociodemographic characteristics, history of pregnancy and adverse pregnancy outcomes, physical conditions, living habits during pregnancy). Compared to other exposure groups, the protective effect of starting taking FA at least 3 months before the last menstrual period was stronger (Fig. 2). However, among women who smoked during pregnancy, women who took FA during periconception had a higher risk of preterm birth than women who did not take FA (aOR 1.80–2.52), but the results were not statistically significant (*P* > 0.05).

## Discussion

Preterm birth is a global public health. It is estimated that the global preterm birth rate in 2014 was about 10.6% (9.0–12.0%), equating to an estimated 14.84 million live preterm births, of which the preterm birth rate in Asia was about 10.4% [20]. China has the second greatest number of preterm births in the world, and about 7.8% of global preterm births occur in China, second only to India [8]. In this large cohort of 201,477 women in China, we found that FA supplementation before conception was associated with reduced risk of preterm birth. Compared with women who did not take FA, women who started taking FA 1–2 months before their last menstrual period had a 15% lower risk of preterm birth (aOR = 0.85, 95% CI 0.79–0.92), and women who started taking FA at least 3 months before their last menstrual period had a 20% lower risk of preterm birth (aOR = 0.80, 95% CI 0.75–0.87), but women who started taking FA after their last menstrual period did not appear to reduce the risk of preterm birth (aOR = 0.95, 95% CI 0.88–1.03). According to different baseline characteristics, the associations were consistent in different subgroups.

Previous studies have shown inconsistent associations between FA supplementation during periconception and preterm birth. The 1999–2012 Jiaxing Birth cohort showed that among 240,954 women, FA supplementation was associated with lower risk of preterm birth, and the association was only significant in pre-conceptional FA supplementation (OR = 0.92, 95% CI 0.85–1.00) [21]. A case–control study conducted among Sudanese women in 2015 showed that the median (interquartile) level of FA was significantly lower in the 56 cases (preterm birth) than the level in the 56 controls (4.8 ng/ml vs 9.5 ng/ml) [22]. A birth cohort study conducted between 2010 and 2012 in Lanzhou showed that compared to non-users, FA supplement users had a reduced risk of preterm birth (OR = 0.80, 95% CI 0.68–0.94) [23]. The significant reduced risk was mainly observed for those who had used FA supplements for more than 12 weeks (OR = 0.67, 95% CI 0.55–0.83) with a significant dose–response (P for trend = 0.01). After stratifying by starting time of FA use, significant associations were observed for those who took supplements during both preconception and pregnancy (OR = 0.75, 95% CI 0.61–0.92) or during pregnancy only (OR = 0.82, 95% CI 0.69–0.97) [23].

Some studies have not found an association between FA supplementation and preterm birth in women. A prospective cohort study in America in 2015 involving 3,647 women found no association between FA supplementation and preterm birth (*P* > 0.05) [3]. A prospective cohort study in Norway was to examine the association of dietary folate intake and FA supplementation during different periods of pregnancy with the risk of spontaneous preterm delivery and showed that the amount of dietary folate intake (HR 1.00; 95% CI 0.61–1.65) and supplemental folate intake (HR 1.00; 95% CI 1.00–1.00) was not significantly associated with the risk of preterm birth among 66,041 women, and the initiation of FA supplementation more than 8 weeks before conception was associated with an increased risk for spontaneous preterm birth (HR 1.18; 95% CI 1.05–1.32) [24]. A systematic review and meta-analysis of randomized controlled trials in 2016 included 5 trials and showed that women who received FA supplementation had a similar rate of preterm birth < 37 weeks (22.6% vs 22.9%, RR 0.99, 95% CI 0.82–1.18) [25]. The association between FA supplementation and preterm birth has been inconsistent in different studies. This may be due to differences in the dose of FA, pre- or postconceptional beginning and end of supplementation, use of multivitamins, length of supplementation, and the diagnostic criteria for preterm birth [26]. This association may also differ due to different ethnic and preterm birth rates in different countries, and there may be confounding factors in observational studies that have not been taken into account.

The period around conception (2–3 months before and after) is a critical period for optimizing gamete function, and early placental development [7]. Epidemiological data and findings from developmental biology suggest that intervening to improve women’s nutritional status before pregnancy improves long-term outcomes for mothers and babies [27]. The causes of preterm birth are complex and the exact biological mechanisms underlying a putative effect of higher FA concentrations on lower preterm birth risk are not clear. The demand for FA increases during pregnancy due to rapid maternal and fetal cellular growth and development. FA has an essential role in DNA methylation and synthesis [2]. A low folate concentration may perturb mitotic cell division which may affect the development of the placenta [28]. FA may also affect placenta implantation and vascular remodeling through its role as a superoxide scavenger in antioxidant defenses [28]. In addition, higher FA concentrations may also reduce preterm birth risk by conferring protection against intrauterine infection [29]. Further study on the underlying mechanisms is required.

Our study also found that periconceptional FA supplementation could reduce the incidence of early preterm birth (gestation less than 34 weeks). Women who started taking FA after their last menstrual period, 1–2 months before their last menstrual period, and at least 3 months before their last menstrual period had a 14%, 19%, and 23% lower risk of preterm birth than women who did not take FA (*s* < 0.05). Similar results were found in the Lanzhou birth cohort, where FA supplementation was associated with a 50% reduction in early preterm birth compared to women who did not take FA [23]. Early preterm birth is usually caused by intrauterine infection and inflammation and most deaths from preterm birth are concentrated in early preterm birth [30].

A large number of randomized trials have shown that supplementation with FA during periconception can prevent NTDs in newborns [31, 32]. As a result, periconceptional FA supplementation is recommended by health authorities in many countries around the world. Chinese Ministry of Health recommends that women of childbearing age take 0.4 mg of FA tablets every day from at least three months before conception until their first trimester of pregnancy [33]. Our study found that FA supplementation before and during conception is a protective factor for preterm birth, particularly if FA were taken at least 3 months before conception. Therefore, supplementation with FA during periconception can reduce the risk of preterm birth, thereby reducing neonatal mortality and ensuring maternal and child health.

Our study has several strengths. First, this study was a large population-based retrospective cohort study that recruited all women who participated in the NFPHEP and had a singleton livebirth from 129 counties of Southwest China between 2010 and 2018, which may have less selection bias. The information about exposure in this study was collected during the follow-up in their first trimester of pregnancy, thus reducing the recall bias as much as possible. In addition, this was the first explorative study of periconceptional FA supplementation and preterm birth in a large cohort in Southwest China. The cohort included more than 40 ethnic minorities living in the region, and our results are more representative of the multi-ethnic situation in China than other studies. However, our study has some limitations. The NFPHEP did not collect information on pregnancy complications such as gestational hypertension, gestational diabetes and, therefore, may influence the interpretation of the results to some extent.

## Conclusions

In this retrospective cohort study of 200,000 Chinese women, periconceptional supplementation with FA was associated with a lower risk of preterm birth. Women who started taking FA at least 3 months before their last menstrual period were more likely to reduce the risk of preterm birth than women who started taking FA 1–2 months before their last menstrual period.
